# Social Coordination Information in Dynamic Chase Modulates EEG Mu Rhythm

**DOI:** 10.1038/s41598-017-04129-2

**Published:** 2017-07-06

**Authors:** Jun Yin, Xiaowei Ding, Haokui Xu, Feng Zhang, Mowei Shen

**Affiliations:** 1 Department of Psychology, Ningbo University, Ningbo, P.R. China; 2 Department of Psychology and Behavioral Sciences, Zhejiang University, Hangzhou, P.R. China

## Abstract

Understanding actions plays an impressive role in our social life. Such processing has been suggested to be reflected by EEG Mu rhythm (8–13 Hz in sensorimotor regions). However, it remains unclear whether Mu rhythm is modulated by the social nature of coordination information in interactive actions (i.e., inter-dependency). This study used a novel manipulation of social coordination information: in a computer-based task, participants viewed a replay of two chasers chasing a common target coordinately (coordinated chase) or independently (solo chase). Simultaneously, to distinguish the potential effect of social coordination information from that of object-directed goal information, a control version of each condition was created by randomizing one chaser’s movement. In a second experiment, we made the target invisible to participants to control for low-level properties. Watching replays of coordinated chases induced stronger Mu suppression than solo chases, although both involved a common target. These effects were not explained by attention mechanisms or low-level physical patterns (e.g., the degree of physical synchronization). Therefore, the current findings suggest that processing social coordination information can be reflected by Mu rhythm. This function of Mu rhythm may characterize the activity of human mirror neuron system.

## Introduction

Human beings do not always pursue one’s own individual goals, but often interact with each other to achieve a collective/shared goal^1, 2^. For this form of interaction, two or more individuals coordinate with their actions to collectively affect their environment; this structure informs cooperative activity in humans^3, 4^. Making sense of such coordinated actions from a third-person perspective constitutes an essential part of our social life and poses an impressive role for further social-cognitive processing (e.g., moral judgment, constructing reputation)^5–7^; however, the neural signals involved in social interpretation of coordinated actions remain largely unidentified.

Action understanding has been extensively shown to be reflected by Mu rhythm (suppression), which is an EEG oscillation of 8–13 Hz mainly distributed over the sensorimotor regions^8–14^. Mu rhythm has been proposed to reflect the activity of human mirror neuron system (MNS), including inferior parietal lobule (IPL) and inferior frontal gyrus (IFG), which serves as one of important neural substrates in understanding actions and goals^15–17^. Though the relation between Mu rhythm and MNS is still under debate, the function of Mu rhythm related to action understanding has been well documented^18, 19^. Gastaut and Bert first reported that Mu rhythm was suppressed during executing active movements as well as observing others’ actions^8^. These results were further confirmed by later EEG and MEG studies^20, 21^. Researchers even found that degraded images of action based on point-light biological motion can modulate Mu rhythm^14^. Moreover, a growing body of studies suggested that Mu rhythm is sensitive to various parameters of actions, such as forms, directness, and the values associated with actions^9, 22–24^. For example, a transitive action with a directed goal suppressed the Mu rhythm more strongly than an intransitive action^9, 23^. Additionally, observing rewarding actions suppressed the Mu rhythm more than punishing or neutral actions did^24^.

The role of Mu rhythm has recently been extended to understanding social interaction^11, 25, 26^. For example, Oberman and colleagues found that the degree of social interaction (e.g., non-interacting: three individuals tossing a ball up in the air to themselves; interacting: three individuals tossing a ball to each other) modulated the amplitude of Mu rhythm^11^. Similarly, Perry and colleagues used the Rock–Scissors–Paper game to show that the social interactive context of the motion affected Mu suppression^25^. Findings from online social interaction also support this conclusion^27–29^. In previous studies, two or more persons usually used different gestures to manipulate the degree of social interaction (e.g., tossing a ball individually or interactively, tapping fingers coordinately or solely), or they did not use coordinated actions (e.g., Rock–Scissors–Paper game). Regarding such social interaction manipulations, some low-level physical features may explain social interaction’s effect on Mu rhythm (e.g., physical fit between gestures, physical synchronization between participants). Even if these physical factors are well controlled, it remains unclear if Mu rhythm is sensitive to the specifically social nature of inter-dependency of coordinated actions (i.e., to social coordination information) or merely to interacting agents’ possession of a common goal. Therefore, this study investigated whether social coordination information processing affects Mu rhythm.

This study used two dynamic chase conditions to manipulate coordination information, as follows. In a computer-based task, two agents chased a common “prey” in either a coordinated or a solo manner (henceforth, “interactive action” refers to actions occurring between the chasers, whereas “object-directed action” refers to actions between a chaser and the target). These chase conditions followed Heider and Simmel^30^, who used geometric figures in a chasing motion. Regarding such display, the figures’ movement constituted the only source of socially significant information. Here, we utilized man-made trajectories to present chase scenes, because the principles of movement remain unclear regarding multi-agent chasing with two chasers and one target^31^. This design granted control over possible confounding factors in typical interaction scenes by dissociating the included social coordination information from the psychological commonness of the chasers’ goals, and thereby permitting differentiation of those factors’ respective effects on the Mu rhythm. This was possible because the agents in both chase configurations pursued a common goal but only engaged in socially interactive action in the coordinated configuration. This design also permitted exclusion of the possible effect of physical correlation between pursuers in the coordinated condition by making comparison of Mu suppression when the target was visible and when it was not visible to the viewer. This was effective because both settings involved two chasers but only the latter setting restricted interaction information^32, 33^.

In this context, if Mu rhythm had its role in understanding interaction goals and can reflect social coordination information, we should observe the difference in Mu suppression between watching coordinated and solo chases after controlling for goal-directed information (Experiment 1). Since these two chases differ regarding social coordination information, and that any such difference would disappear if social coordination information was absent, although both chasers retained the same physical characteristics as in Experiment 1 (i.e., concealing the target; Experiment 2). Moreover, because when watching coordinated actions, the observer treats the involved individuals as a unit due to their inter-dependency^32, 33^ and accordingly, the humans need to simultaneously understand more actions^34, 35^, challenging the requirement for generating Mu rhythm. Therefore, we additionally predicted that coordinated interaction between two chasers would elicit greater Mu suppression than solo chases.

## Experiment 1

Participants viewed replays of coordinated and solo chases; however, direct comparison of Mu activation between these conditions could not exclude physical properties’ possible effects. Therefore, we introduced a control for each chase condition: one chaser in the replay was replaced with a randomly moving agent, whereas the remaining chaser still chased the objective. We calculated Mu suppression compared with this control condition separately for the coordinated and solo chases, and then compared the resulting difference values between the conditions. In this case, Mu suppression would reflect the functional induction by the social interaction information between the two chasers after controlling the object-directed goal information. Therefore, the differences in Mu suppression under such comparison should be attributed to the experienced coordination information between the two chasers.

## Methods

### Participants

Participants were 24 students from Zhejiang University (16 men, 9 women; age: 18–28 years). All participants reported normal or corrected-to-normal vision and normal color vision. None reported a history of neurological disorder. This study was approved by the Research Ethics Board of Zhejiang University and granting agency, and was performed in accordance with the relevant guidelines and regulations. All participants received information sheets about the experimental procedure and signed informed consent forms after learning the purpose and the procedure of the experiment.

### Stimuli

Four types of chasing motions with three agents were used here. The trajectories of chasing motions were either from recorded data as real-world humans controlled their own avatars in computers with a coordinated/solo chase toward the same target, or from modified motions through adjusting trajectories of one agent.

The movement trajectories were recorded according to the following steps: Three participants formed a group and were asked to take part in a chasing game and sat without head restraint approximately 60 cm from a monitor (the measurements were computed based on this viewing distance). Each group member controlled an agent: one played the role of prey by controlling a red square (1° × 1°) on the screen with a mouse, while the other two played the role of predators by controlling green and blue discs of 1° diameter on the screen. The two predators were required to chase the common prey, either in a coordinated (i.e., cooperative interaction) or solo (i.e., capturing the target by yourself with minimum or without interaction) manner, and the prey tried to avoid being caught. If any predator reached the prey, the trial ended. To prevent the prey from being caught at the beginning, the initial distances between each pair of agents were greater than 5°. Participants could move the agents less than 0.5°/frame and the controlled agents could not pass each other according to the algorithm that each agent cannot occupy the same space of the remaining agents on the screen; they only controlled their own agents within a commonly limited zone bounded by a visible gray square (25° × 25°), whereas the monitor subtended 36.6° × 27.6°. This chasing game was executed on PC monitors (resolution: 1024 × 768; refresh rate: 60 Hz) using custom software written in MATLAB with the Psychophysics Toolbox libraries^36^. Each group member controlled a PC and saw the same online chasing motions on the screen; the online positions were transferred between PCs by the TCP/IP protocol. Their dynamic positions were recorded. Finally, five trajectories each were obtained for the coordinated and solo chases, whose durations ranged from 4–10 s; the last 1 s of each recording was discarded, since the three agents usually tangled before the prey was caught. We produced 10 new trajectories by adding intermediate positions between each pair of adjacent frames using the mean of the two positions to generate smoother trajectories with longer durations. These two types of recorded trajectories were named “Original-Coordinated” and “Original-Solo,” respectively.

**Figure 1:**
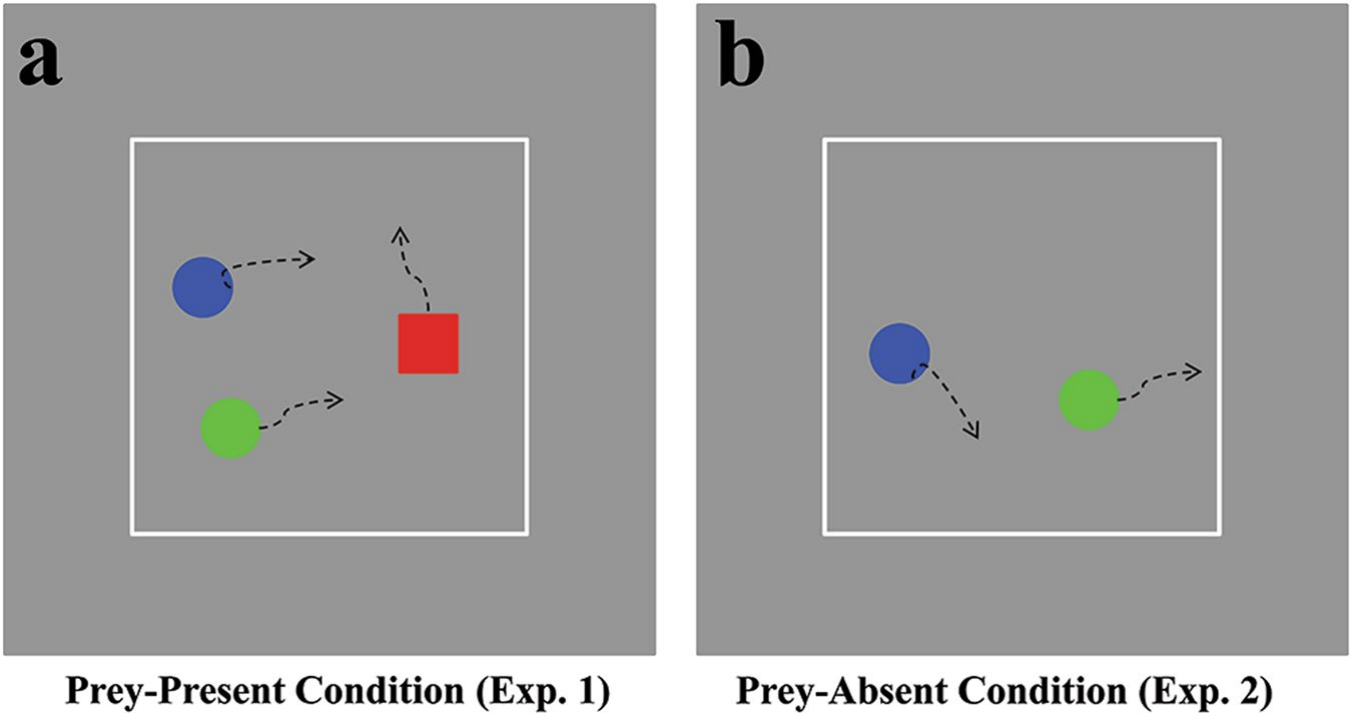
An illustration of chasing motion in two experiments. (**a**) and (**b**) Depict a sampled image from the dynamic display lasting 3 s in Experiment 1 and Experiment 2, respectively. The curved dashed arrows represent each agent’s motion; these were not present during trials.

We made systematic changes to the trajectories to create control versions of them. These control trajectories were necessary in order to permit exclusion of object-directed goal information’s possible effect on Mu rhythm; they were as follows. (1) Modified-Coordinated: we computed the mean velocities in each Original-Coordinated trajectory for each agent (i.e., the “human velocity”). The trajectory of one disc was subsequently replaced with Brownian motion of similar constraints as the real human trajectory (i.e., the chaser’s velocity was centered on the mean human velocity within ±5°/s of movement). Finally, we selected new trajectories whose average distance between the original and modified chaser agent equaled the average distance between the chasers in the original human trajectory. (2) Modified-Solo: we generated this trajectory using the same protocol as the Modified-Coordinated trajectory, except using the Original-Solo trajectories. Hence, ten trajectories for each control were produced in accordance with this procedure, since each trial included two chasers (Fig. 1; see also the supplementary videos). Our previous studies^33^ and pilot investigation confirmed that participants could extract the coordinated interaction information as we expected, except as discussed below. When more than one chaser is running, it is nearly impossible for an individual to be completely alone in the chase toward the same target. For instance, one agent occupies the possible positions of others, and to some extent, exhibits illusory competitive chasing, which was observed in our study. Therefore, “solo chasing” indicates that the agents chased their goal without coordinated interaction, or at least with less coordination, than in coordinated chasing.

Owing to the new dynamic trajectories’ velocities were lower, the trajectories were presented using a 70 Hz refresh rate in the replay stage. The replays consisted of an excerpt lasting 3 s that was randomly segmented from the recorded trajectories. Additionally, the replayed positions were rotated randomly by 0°, 90°, 180°, or 270° around the center of the screen.

### Procedure and design

In each trial, a pink dot subtending 0.5° of visual angle was first presented at the center of the screen for 0.4–0.5 s in a restricted zone identical to that used during the recording stage (dot color: 255, 0, 255, RGB). This dot indicated to the participants that the recording would begin. Subsequently, one disc would flash white for 3 s. Participants reported the color of the flashing disc (i.e., blue or green) as accurately as possible by pressing the “F” or “J” key with their left or right index finger, respectively. The trial interval’s duration was 1.5–2 s.

Four types of trajectories could be treated as a combination of 2 (chase type: coordinated vs. solo) × 2 (trajectory source: original vs. modified) within-subject design. Each type of trajectories had 80 trials, resulting in 320 trials in total, which were presented randomly. For participant’s behavioral responses, reaction times (RTs) and accuracies were recorded to check the task difficulty.

### Electrophysiological recording and analysis

Participants’ EEG was recorded from 32 scalp sites using Ag/AgCl electrodes mounted in an elastic cap (Neuroscan Inc., USA). All recordings were referenced to the left mastoid. Vertical electrooculogram (VEOG) and horizontal electrooculogram (HEOG) were recorded with two pairs of electrodes, one pair placed above and below the left eye, and the other pair placed beside the two eyes. All inter-electrode impedances were maintained below 5 kΩ. The EEG and EOG were amplified using SynAmps with a 0.05–100 Hz bandpass and continuously sampled at 500 Hz/channel for off-line analysis.

Data were analyzed using Neuroscan software and Matlab’s Fieldtrip toolbox^37^. Raw data were initially re-referenced offline to the average of the left and right mastoids, and then digitally filtered offline with a 0.1–40 Hz (24 dB/oct) bandpass filter. Electrooculogram artifacts were corrected via the regression method^38^. Additional artifact rejection was applied to epochs with EEG amplitude exceeding ±100 μV. The EEG was segmented into 3000-ms epochs starting from the onset of motion and including the whole moving time. For each epoch, the integrated power in the 8–13 Hz range was computed through a Fast Fourier Transform (FFT) performed at 1/3 Hz intervals (using a Hanning window). Segments’ absolute power was subsequently averaged for each channel, each condition, and each subject. Regarding the dependent variable, we did not use the absolute power but computed the logarithm of power ratio between the Original-Coordinated and Modified-Coordinated trajectory conditions and between Original-Solo and Modified-Solo trajectory condition, since each of the replaced trajectory conditions were treated as the baseline to get pure effect from the social interaction information. This method is commonly adopted, because (1) it corrects for variability in the absolute power as a result of individual differences, such as scalp thickness, electrode placement, and impedance^11, 25^, and (2) the log transform for ratio is aimed to meet with the requirements of normal distribution (this yielded a “suppression index”; named the Mu or Alpha index as appropriate). Finally, participants’ Mu index was computed at C3 and C4 because the Mu rhythm was assumed to emerge at the sensorimotor regions corresponding to those electrodes’ location in the 10–20 system, and because previous research examining action understanding has typically used these sites^11, 24, 25^. Additionally, the Mu signal oscillates with the same frequency as the posterior Alpha signal, which is mainly located at the occipital site and is sensitive to changes in attentional state^39, 40^. Therefore, occipital sites O1 and O2 were selected to measure Alpha modulation (Alpha index) and determine if spreading of attention-related Alpha rhythm underlies social coordination information’s effect on Mu rhythm. Further, to test whether each of Modified-Solo and Modified-Coordinated conditions as relative baselines induce Mu suppression, a common baseline of resting state between blocks was adopted and the logarithm of power ratio between Modified-Coordinated trajectory/Modified-Solo trajectory condition and this common baseline was computed. The negative value would indicate the occurrence of Mu suppression in our used relative baselines.

## Results

### Behavioral Results

**Figure 2:**
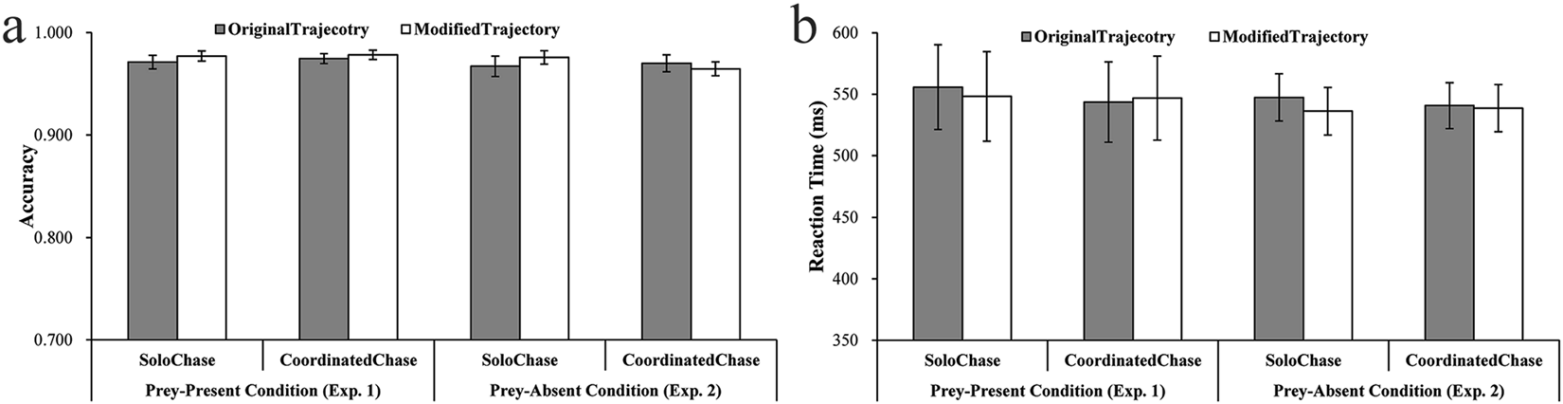
Behavioral results of judging the color of the flashed disc. (**a**) and (**b**) Show accuracies and reaction times (RTs) of each condition, respectively. Error bars indicate standard errors (±SE).

Figure 2a and b show the accuracies and RTs for all conditions (please see Table 1 for descriptive statistics). A two-way ANOVA (Analysis of Variance) taking the chase type and trajectory source as two factors was conducted on RT and accuracy, respectively. There was no effect of chase type [accuracy: *F*(1, 23) = 0.39, *p* = 0.54, η_*p*_^2^ = 0.02; RT: *F*(1, 23) = 1.47, *p* = 0.24, η_*p*_^2^ = 0.06] or trajectory source [accuracy: *F*(1, 23) = 2.00, *p* = 0.17, η_*p*_^2^ = 0.08; RT: *F*(1, 23) = 0.30, *p* = 0.59, η_*p*_^2^ = 0.01], nor any interaction between the two [accuracy: *F*(1, 23) = 0.07, *p* = 0.79, η_*p*_^2^ < 0.01; RT: *F*(1, 23) = 2.24, *p* = 0.15, η_*p*_^2^ = 0.09], found for either accuracy or RT. These results indicate that task difficulty was consistent between conditions; therefore, task difficulty was excluded from possibly underlying any observed effect on the Mu and Alpha indexes.

**Table 1:** Accuracy and reaction times (ms) for all conditions (mean ± SE).

		Solo Chase		Coordinated Chase	
		Original Trajectory	Modified Trajectory	Original Trajectory	Modified Trajectory
Exp. 1	Accuracy	0.971 ± 0.007	0.977 ± 0.005	0.975 ± 0.005	0.978 ± 0.005
	RT	556 ± 34	548 ± 37	544 ± 33	547 ± 34
Exp. 2	Accuracy	0.967 ± 0.010	0.976 ± 0.007	0.970 ± 0.008	0.965 ± 0.007
	RT	547 ± 19	536 ± 19	541 ± 19	539 ± 19

### EEG results

**Figure 3:**
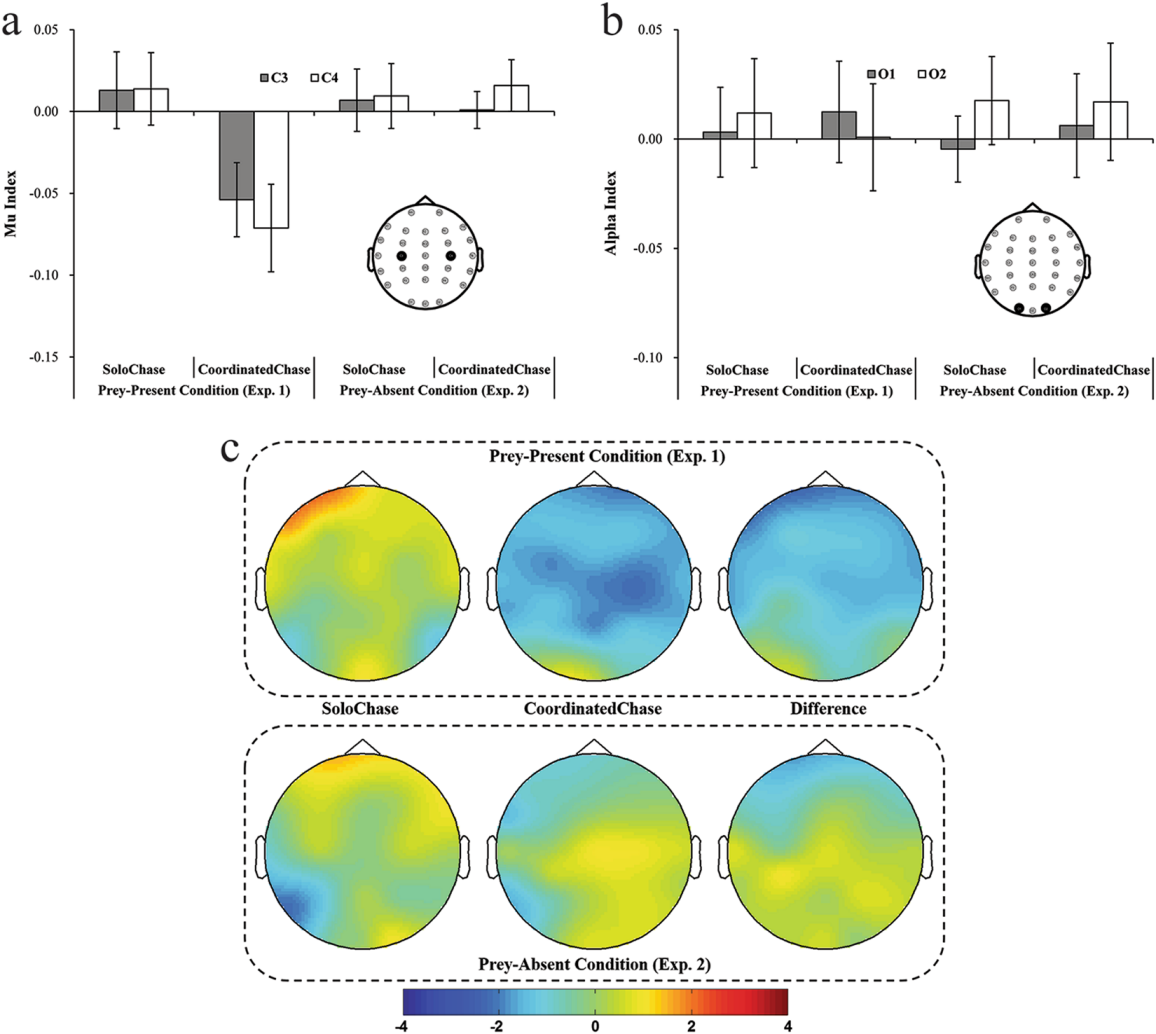
EEG results when watching different kinds of chasing actions. (**a**) and (**b**) Show the patterns of Mu index and Alpha index, respectively. (**c**) Depicts topographic representations for t- tests against zero of each condition and for t-tests for differences between both conditions (i.e., the coordinated chase condition relative to the solo chase condition). The color in (**c**) represents t-tests values against zero in figures of two left columns and t-tests for differences between the coordinated chase condition and the solo chase condition in figures of one right column. Error bars indicate standard errors (±SE).

The topographical distribution of t-test values indicated that chase type modulated Mu index but not Alpha index (Fig. 3c, Table 2). To confirm this observation, a 2 (scalp location: central, occipital) × 2 (hemisphere: left, right) × 2 (chase type: coordinated, solo) three-way ANOVA was conducted on suppression index. This analysis yielded a marginally significant main effect of scalp location [*F*(1, 23) = 3.82, *p* = 0.06, η_*p*_^2^ = 0.14], showing that 8–13 Hz rhythm is tended to be suppressed stronger in central C3 and C4 sites; a significant interaction effect between chase type and scalp location [*F*(1, 23) = 4.65, *p* = 0.04, η_*p*_^2^ = 0.17]. The simple effect test following this interaction effect indicated greater Mu suppression at central sites in the coordinated chase condition than in the solo chase condition (*p* = 0.001), but indicated no significant Alpha index difference at the occipital sites between the coordinated and solo chase conditions (*p* = 0.84). No significant effects were identified regarding either of the left main effects [hemisphere: *F*(1, 23) = 0.17, *p* = 0.68, η_*p*_^2^ < 0.01; chase type: *F*(1, 23) = 2.61, *p* = 0.12, η_*p*_^2^ = 0.10] or the other interaction effects [scalp location × hemisphere: *F*(1, 23) = 0.18, *p* = 0.67, η_*p*_^2^ < 0.01; hemisphere × chase type: *F*(1, 23) = 1.19, *p* = 0.29, η_*p*_^2^ = 0.05; scalp location × hemisphere × chase type: *F*(1, 23) < 0.01, *p* = 0.95, η_*p*_^2^ < 0.01]. In addition, compared to its control condition baseline, the coordinated chase condition exhibited Mu suppression (i.e., Mu suppression was significant from zero; C3: *t*(23) = 2.38, *p* = 0.026, Cohen’s d = 0.49; C4: *t*(23) = 2.66, *p* = 0.014, Cohen’s d = 0.54; other tests: *t*s < 0.65, *p*s > 0.50]. Additionally, compared with the common resting state baseline (i.e., by pooling C3 and C4’s results), both relative baselines exhibited Mu suppression (Modified-Solo: *t*(23) = 11.92, *p* < 0.001, Cohen’s d = 2.43; Modified-Coordinated: *t*(23) = 11.78, *p* < 0.001, Cohen’s d = 2.40). These results suggest that chases with social coordination information (i.e., the coordinated chase conditions) elicited greater Mu suppression than the solo chases. Social coordination information did not modulate the Alpha index (which indicates attentional involvement), excluding the possibility that an attention component underlay social coordination information’s effect on the Mu rhythm.

**Table 2:** Mu and Alpha indices for all conditions (mean ± SE).

	Index	Solo Chase		Coordinated Chase	
		Left Hemisphere (C3 or O1)	Right Hemisphere (C4 or O2)	Left Hemisphere (C3 or O1)	Right Hemisphere (C4 or O2)
Exp. 1	Mu	0.013 ± 0.023	0.014 ± 0.022	−0.054 ± 0.023	−0.071 ± 0.027
	Alpha	0.003 ± 0.021	0.012 ± 0.025	0.012 ± 0.023	0.001 ± 0.025
Exp. 2	Mu	0.007 ± 0.019	0.009 ± 0.020	0.001 ± 0.011	0.016 ± 0.016
	Alpha	−0.005 ± 0.015	0.018 ± 0.020	0.006 ± 0.023	0.017 ± 0.027

## Experiment 2

Even though subtracting the Mu activation of the modified trajectory from the original trajectory could isolate the effect of coordination contents from goal-directed information, it is accompanied by some minor changes in the low-level motion features, such as the degree of physical synchronization between two chasers, and trajectory differences between the replaced agent and its referred chaser. Therefore, these changes may have caused differences in Mu modulation observed in Experiment 1. Experiment 2 tested this possibility with a new design: in all conditions, the chase scenes were presented with the prey made invisible and only the chasers visible. The chasers’ behavior responded continuously to that of the prey; therefore, if the prey is concealed, an observer may experience more difficulty determining the chasers’ intent, although their movements are unaltered relative to the original chase scenes^32, 41^. This design weakened the viewer’s impression of social coordination but retained the trajectory differences introduced in Experiment 1 following the manipulation of the original chaser trajectory and did not change the degree of physical synchronization. Therefore, if the new design eliminated the observed difference in Mu activation, this would suggest that Experiment 1’s results were owing to social coordination information; otherwise, the results would be attributed to the introduced differences in low-level motion characteristics.

## Methods

This study was approved by the Research Ethics Board of Zhejiang University and granting agency, and was performed in accordance with the relevant guidelines and regulations. Participants were 24 naïve Zhejiang University students (11 men, 13 women; age: 18–27 years). The experimental method was identical with Experiment 1, except that the prey (i.e., the red square) was not displayed in the recorded chases.

## Results and Discussion

### Behavioral Results

Figure 2a and b depict the overall accuracies and RTs for different types of trajectory (please see Table 1 for descriptive statistics). Taking chase type and trajectory source as factors, a two-way ANOVA was conducted on accuracy [chase type: *F*(1, 23) = 0.65, *p* = 0.43, η_*p*_^2^ = 0.03; trajectory source: *F*(1, 23) = 0.08, *p* = 0.78, η_*p*_^2^ < 0.01; chase type × trajectory source: *F*(1, 23) = 1.53, *p* = 0.23, η_*p*_^2^ = 0.06] and RT [chase type: *F*(1, 23) = 0.17, *p* = 0.68, η_*p*_^2^ < 0.01; trajectory source: *F*(1, 23) = 3.05, *p* = 0.09, η_*p*_^2^ = 0.12; chase type × trajectory source: *F*(1, 23) = 0.86, *p* = 0.36, η_*p*_^2^ = 0.04], respectively, showing that no significant result was found. These results suggest that task difficulty was consistent across conditions and therefore excluded the possibility that task settings underlay any observed index modulation.

### EEG results

As shown at Fig. 3c, the differences in the Mu index found in Experiment 1 vanished accordingly when the prey was absent although the Alpha index retained the same pattern as Experiment 1 (Fig. 3a; Table 2). This observation was confirmed by three-way ANOVAs with scalp location, hemisphere and chase type within-subject factors. Specifically, we did not find any significant result in either main effects [scalp location: *F*(1, 23) < 0.01, *p* = 0.96, η_*p*_^2^ < 0.01; hemisphere: *F*(1, 23) = 2.99, *p* = 0.10, η_*p*_^2^ = 0.12; chase type: *F*(1, 23) = 0.02, *p* = 0.90, η_*p*_^2^ < 0.01] or interaction effects [scalp location × hemisphere: *F*(1, 23) = 0.19, *p* = 0.67, η_*p*_^2^ < 0.01; scalp location × chase type: *F*(1, 23) = 0.03, *p* = 0.86, η_*p*_^2^ < 0.01; hemisphere × chase type: *F*(1, 23) < 0.01, *p* = 0.98, η_*p*_^2^ < 0.01; scalp location × hemisphere × chase type: *F*(1, 23) = 0.86, *p* = 0.36, η_*p*_^2^ = 0.04]. Single-sample t-tests for each condition at each electrode relative to zero identified no significant differences (*t*s < 1.01, *p*s > 0.30), suggesting that only the physical variation of the coordinated chase condition did not induce stronger Mu suppression relative to its baseline (i.e., replaying the modified trajectories). Additionally, comparing with the common resting-state baseline by pooling C3 and C4’s results, both relative baselines exhibited Mu suppression (Modified-Solo: *t*(23) = 14.02, *p* < 0.001, Cohen’s d = 2.86; Modified-Coordinated: *t*(23) = 14.08, *p* < 0.001, Cohen’s d = 2.87). These findings indicate that low-level motion differences between the coordinated and solo chase conditions (e.g., differences in chasers’ physical synchronization) did not underlie Experiment 1’s results; otherwise, a similar Mu effect would have appeared, since both experiments used the same pattern of movement between the chasers. Together, these findings indicate that differing social coordination information underlay Experiment 1’s results.

## General Discussion

The present study explored the contributions of Mu rhythm in understanding coordinated interaction. We found that the degree of social coordination reflected in chasing actions modulated Mu rhythm suppression. This effect was not explained by attentional mechanisms or low-level physical characteristics (e.g., the degree of physical synchronization or trajectory differences). Moreover, we found that social coordination induced stronger Mu suppression and was the only condition that elicited Mu suppression relative to its control with goal-directed information. This is consistent with our prediction that social coordination information affects Mu suppression. Therefore, the current data suggest that the social nature of processing social coordination information could be reflected by Mu rhythm.

Differing motor activities of pressing buttons between conditions cannot explain the present findings. In all conditions of each experiment, participants reported the flashing disc figure’s color; importantly, the number and order of correct responses was counterbalanced across conditions. This design permitted exclusion of motor activity from the factors possibly affecting Mu and Alpha activation, while computing the logarithm of power ratio between the coordinated and solo chases (i.e., using the original trajectories) and their corresponding baseline control conditions (i.e., using the modified trajectories). Additionally, Experiments 1 and 2 used the same task setting, but Mu suppression differences between chase conditions were observed in Experiment 1 and not in Experiment 2. This followed concealment of the target (i.e., elimination of social coordination information) but retention of identical chaser trajectories. This comparison also excluded motor activity of pressing buttons from the factors possibly underlying the observed difference in Mu suppression.

This study’s results confirmed previous observations that the occipital-Alpha rhythm and Mu rhythm exhibit characteristically different responses^13, 18, 25^ and supported the suggestion that these signals reflect distinct cognitive functions. It is commonly suggested that the occipital-Alpha rhythm is functional with attentional mechanisms^39, 40^, and Mu rhythm reflects the processing of goals and intentions of observed actions, but the former attentional effects could undermine the second one^18, 19^. This study found no difference in occipital-Alpha activation between coordinated and solo chases after controlling for object-directed goal information, suggesting that engaging in interdependent action and observing it may require equal attention, and excluding the possibility that attentional effects indicated by the Alpha rhythm explain Mu suppression. This finding is consistent with fMRI research showing that regions functional with the recruitment of general attention resources did not activate differently between social interaction and non-interaction, but these two conditions activated differently at regions belonging to MNS^34, 42^, which is indicated to the location to produce Mu rhythm.

This study provided new evidence supporting that the activity of Mu rhythm is linked to our social skills. In previous studies, the role of Mu rhythm had been thought to be in the domain of uncovering the individual goal and intention of observed actions^8–14^. Recently, some studies started to consider the complex social skills of understanding social interaction, and provided initial evidences that Mu rhythm may be involved in it, but still did not specify which factors in the social interaction scenes contributed to the pattern in Mu rhythm^11, 25^. By contrasting the activation between coordinated and solo chases, our research clearly and robustly illustrates that Mu rhythm reflects the processing of social coordination information, which is the key skill to enable us be socially connected. Moreover, in contrast to previous studies, which focused on the behavior actions by humans only^9, 11, 23, 25^, this conclusion is from a brand new context which has no human-alike features, such as body, head, and abstract image (e.g., biological motion). Such finding is in accordance with a recent study showing that actions from a non-anthropomorphic robot induced stronger Mu suppression, if this robot was attributed with more agency (i.e., the more aggressive the action towards the robot) even no human-alike features were included^43^. Therefore, Mu rhythm is sensitive to understanding all actions of animated agents, not just specific to “mirror” actions with human-alike appearance.

The current findings for Mu rhythm may signal the functional properties of human MNS, as it was proposed that mu desynchronization may characterize MNS activity in humans^9, 11, 13, 44, 45^. Similar with Mu rhythm, MNS gets activated during both action execution and observation, and its activation is modulated by the contents of actions^15–17^. One recent meta-analysis evaluated the relation between MNS and Mu rhythm, and concluded that changes in EEG mu activity provide a valid approach to studying human neural mirroring^46^. If this is the case, our research implied that human MNS is involved in processing the social nature of coordinated actions, and more neural computation resource are needed when actions from interactive structure are considered simultaneously. Indeed, recent fMRI studies found interactive actions activates more in regions of mirror network (e.g., IFG) than non-interactive actions^34, 42, 47, 48^, thought their used interaction (e.g., two agents were either face-to-face or the one was turned sideways with respect to the other) could be explained by the factor of physical synchronization. While the examination of the role of MNS on processing coordinated actions is out of scope of current study, it needs further addressing.

In conclusion, this study examined social coordination information’s effect on the Mu and Alpha rhythms. Consistent with our prediction of Mu rhythm reacting more to inter-dependency of coordination actions, we found that processing social coordination information elicited greater Mu suppression than processing similar non-coordinated information, but found no corresponding differential effect regarding the Alpha rhythm. In summary, adding to knowledge of the Mu rhythm’s relationship with individual actions, these findings suggest Mu rhythm suppression can reflect social coordination information processing.

## Electronic supplementary material

Legends of supplementary videos

Video 1

Video 2

Video 3

Video 4

Video 5

Video 6

Video 7

Video 8
