# Supplementary material for: Social Coordination Information in Dynamic Chase Modulates EEG Mu Rhythm
Source: Sci Rep. 2017 Jul 6;7:4782. doi: 10.1038/s41598-017-04129-2 (PMC5500576; doi:10.1038/s41598-017-04129-2)
Supplement: Supplementary file 1 — Legends of supplementary videos [file 41598_2017_4129_MOESM1_ESM.pdf]

Social Coordination Information in Dynamic Chase Modulates EEG Mu Rhythm

Author: Jun Yin[1, 2], Xiaowei Ding[2], Haokui Xu[2], Feng Zhang[1*], Mowei Shen[2*]

[1]Department of Psychology, Ningbo University, Ningbo, P. R. China

[2]Department of Psychology and Behavioral Sciences, Zhejiang University, Hangzhou, P. R. China

***Corresponding author:**

Feng Zhang

Address: Department of Psychology, Ningbo University, No. 616, Fenghua Road, Ningbo, 315211, P. R. China

Telephone: +86-13685891323 (Mobile)

Email: zhangfeng@nbu.edu.cn

and

Mowei Shen

Address: Department of Psychology and Behavioral Sciences, Xixi Campus, Zhejiang University, Hangzhou, 310028, P. R. China

Telephone: +86-13905818750 (Mobile); +86-571-88273820 (Office)

Email: mwshen@zju.edu.cn

Supplementary Information

Here, we provided totally 8 demos for critical conditions. The size of each video frame is 640×480 pixels (the original size in the experiments is 1024×768 pixels). The lasting time for each demo is 3 s with 70 frames per second, which is the same with that in the formal experiments. If you are interested in learning more about the demos and the experiments, please contact with the corresponding author Mowei Shen (mwshen@zju.edu.cn) or the first author Jun Yin (yinjun@zju.edu.cn).

Video 1: A video of replaying original trajectories of coordinated chase with a prey in Experiment 1

Video 2: A video of replaying modified trajectories of coordinated chase with a prey in Experiment 1

Video 3: A video of replaying original trajectories of solo chase with a prey in Experiment 1

Video 4: A video of replaying modified trajectories of solo chase with a prey in Experiment 1

Video 5: A video of replaying original trajectories of coordinated chase without a prey in Experiment 2

Video 6: A video of replaying modified trajectories of coordinated chase without a prey in Experiment 2

Video 7: A video of replaying original trajectories of solo chase without a prey in Experiment 2

Video 8: A video of replaying modified trajectories of solo chase without a prey in Experiment 2
